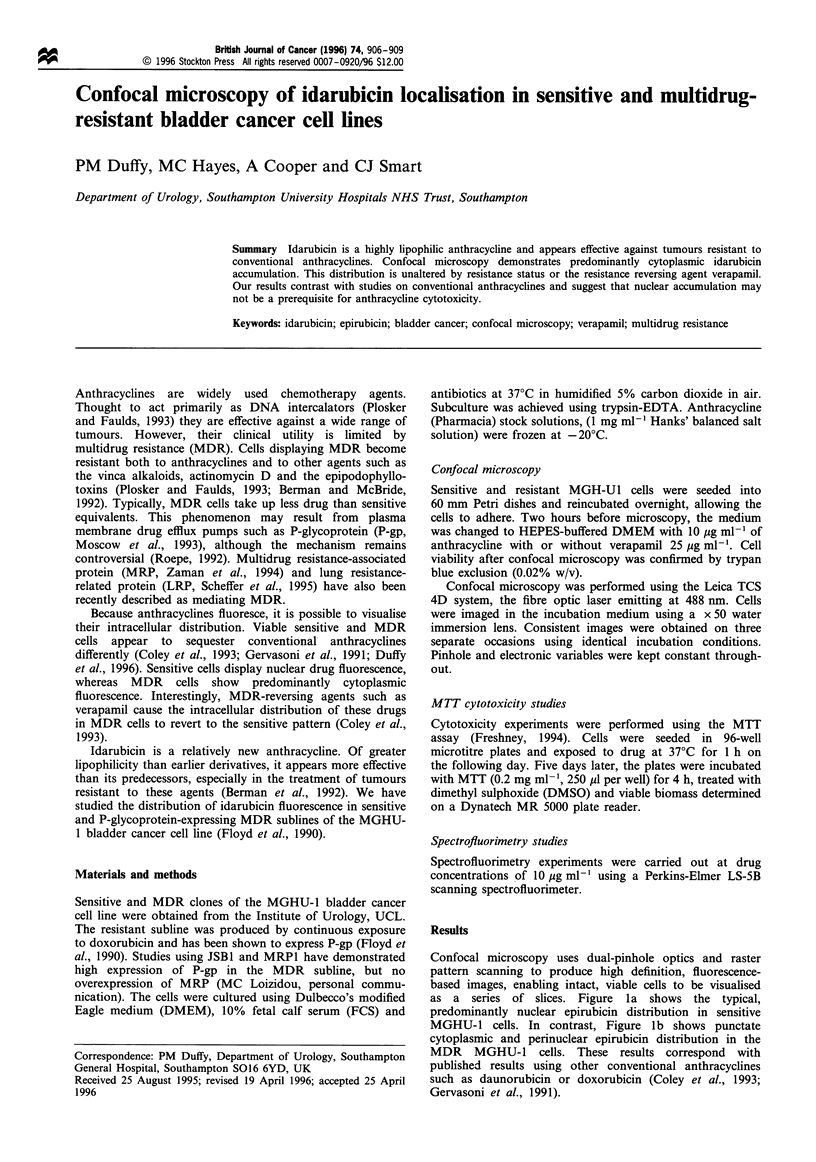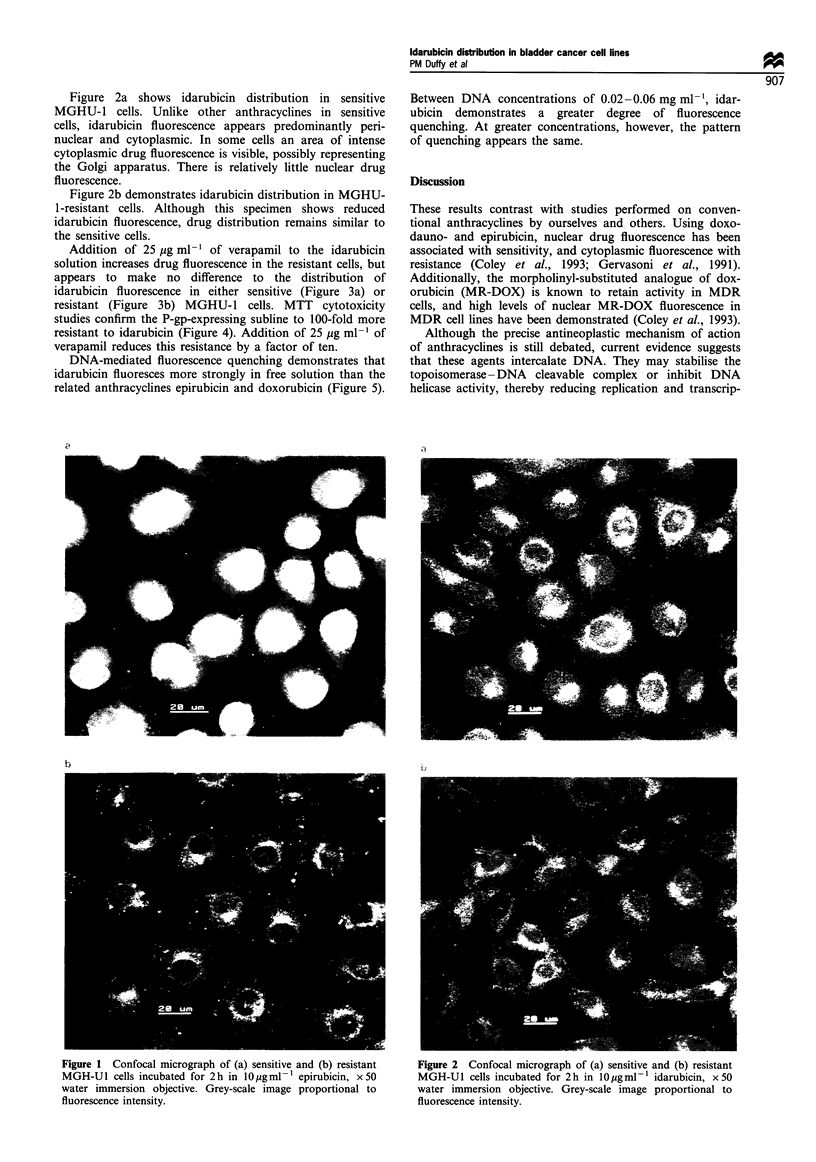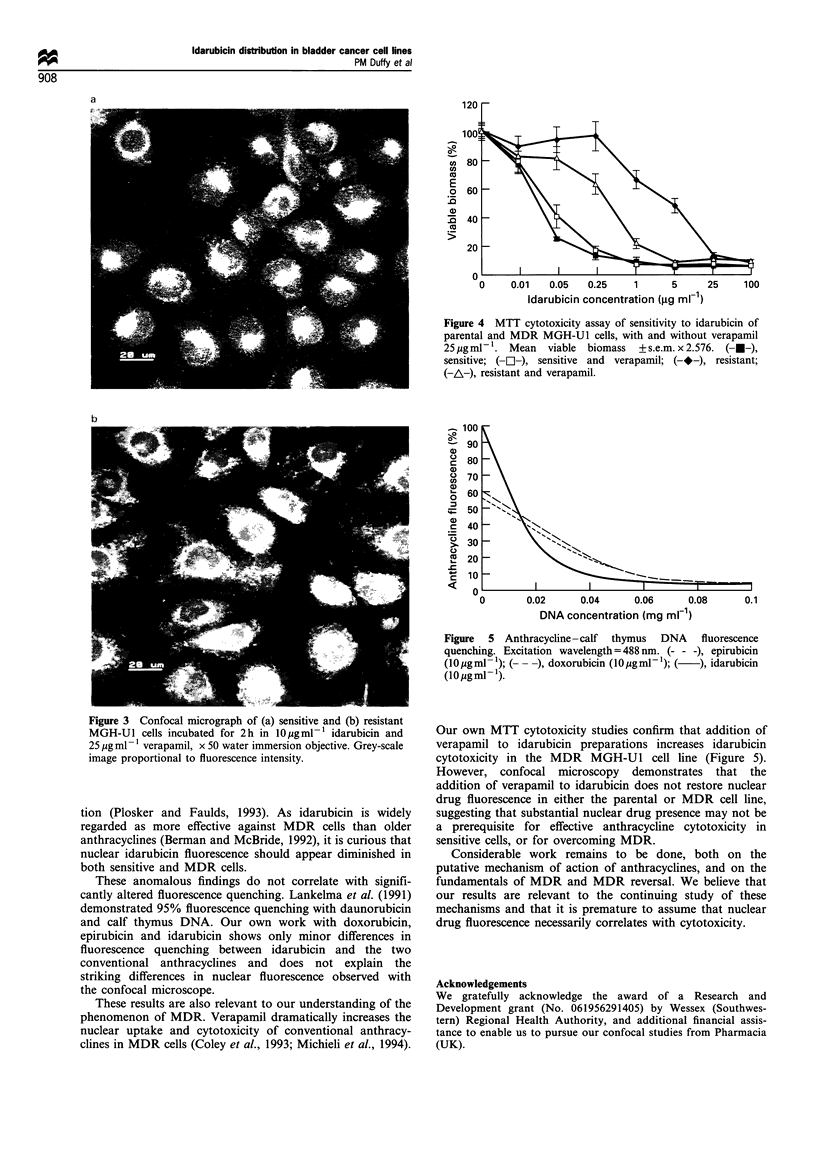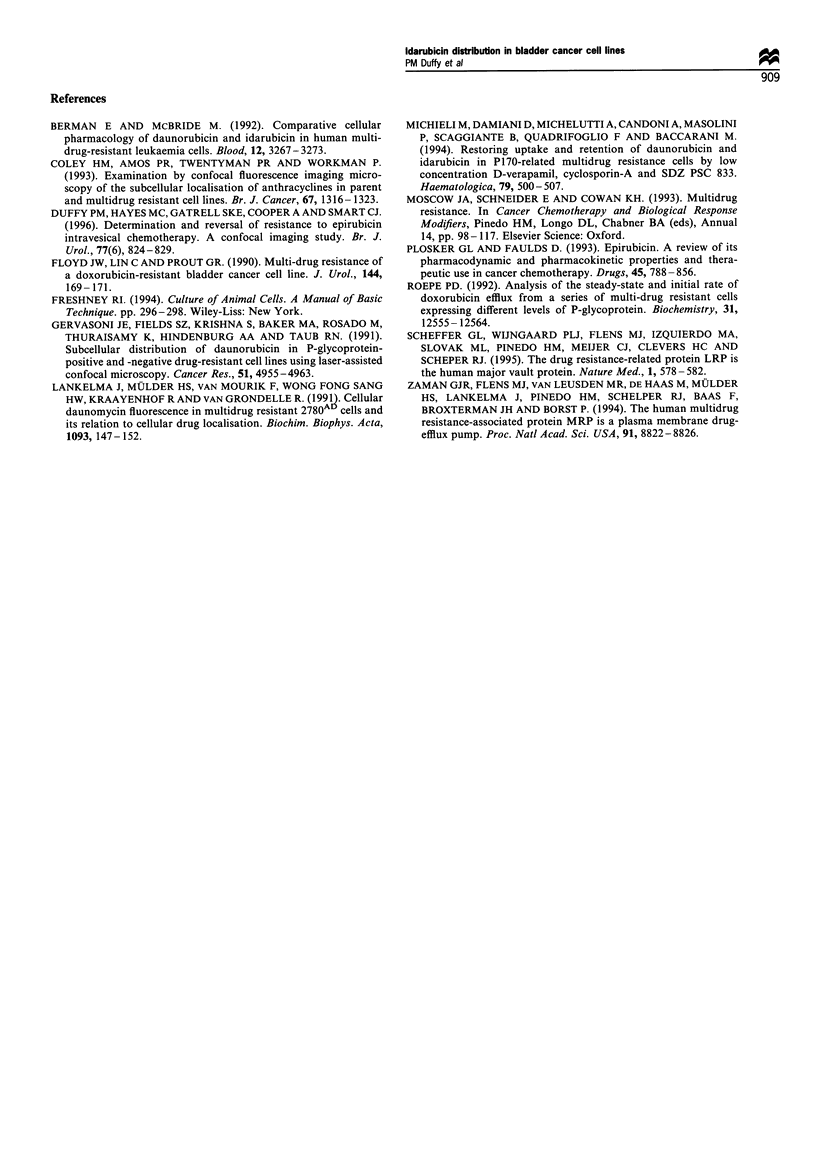# Confocal microscopy of idarubicin localisation in sensitive and multidrug-resistant bladder cancer cell lines.

**DOI:** 10.1038/bjc.1996.455

**Published:** 1996-09

**Authors:** P. M. Duffy, M. C. Hayes, A. Cooper, C. J. Smart

**Affiliations:** Department of Urology, Southampton University Hospitals NHS Trust, U.K.

## Abstract

**Images:**


British Journal of Cancer (1996) 74, 906-909
© 1996 Stockton Press All rights reserved 0007-0920/96 $12.00

### Confocal microscopy of idarubicin localisation in sensitive and multidrug-resistant bladder cancer cell lines

PM Duffy, MC Hayes, A Cooper and CJ Smart

*Department of Urology, Southampton University Hospitals NHS Trust, Southampton*

**Summary** Idarubicin is a highly lipophilic anthracycline and appears effective against tumours resistant to conventional anthracyclines. Confocal microscopy demonstrates predominantly cytoplasmic idarubicin accumulation. This distribution is unaltered by resistance status or the resistance reversing agent verapamil. Our results contrast with studies on conventional anthracyclines and suggest that nuclear accumulation may not be a prerequisite for anthracycline cytotoxicity.

**Keywords:** idarubicin; epirubicin; bladder cancer; confocal microscopy; verapamil; multidrug resistance

Anthracyclines are widely used chemotherapy agents. Thought to act primarily as DNA intercalators (Plosker and Faulds, 1993) they are effective against a wide range of tumours. However, their clinical utility is limited by multidrug resistance (MDR). Cells displaying MDR become resistant both to anthracyclines and to other agents such as the vinca alkaloids, actinomycin D and the epipodophyllotoxins (Plosker and Faulds, 1993; Berman and McBride, 1992). Typically, MDR cells take up less drug than sensitive equivalents. This phenomenon may result from plasma membrane drug efflux pumps such as P-glycoprotein (P-gp, Moscow *et al.*, 1993), although the mechanism remains controversial (Roepe, 1992). Multidrug resistance-associated protein (MRP, Zaman *et al.*, 1994) and lung resistance-related protein (LRP, Scheffer *et al.*, 1995) have also been recently described as mediating MDR.

Because anthracyclines fluoresce, it is possible to visualise their intracellular distribution. Viable sensitive and MDR cells appear to sequester conventional anthracyclines differently (Coley *et al.*, 1993; Gervasoni *et al.*, 1991; Duffy *et al.*, 1996). Sensitive cells display nuclear drug fluorescence, whereas MDR cells show predominantly cytoplasmic fluorescence. Interestingly, MDR-reversing agents such as verapamil cause the intracellular distribution of these drugs in MDR cells to revert to the sensitive pattern (Coley *et al.*, 1993).

Idarubicin is a relatively new anthracycline. Of greater lipophilicity than earlier derivatives, it appears more effective than its predecessors, especially in the treatment of tumours resistant to these agents (Berman *et al.*, 1992). We have studied the distribution of idarubicin fluorescence in sensitive and P-glycoprotein-expressing MDR sublines of the MGHU-1 bladder cancer cell line (Floyd *et al.*, 1990).

### Materials and methods

Sensitive and MDR clones of the MGHU-1 bladder cancer cell line were obtained from the Institute of Urology, UCL. The resistant subline was produced by continuous exposure to doxorubicin and has been shown to express P-gp (Floyd *et al.*, 1990). Studies using JSB1 and MRP1 have demonstrated high expression of P-gp in the MDR subline, but no overexpression of MRP (MC Loizidou, personal communication). The cells were cultured using Dulbecco's modified Eagle medium (DMEM), 10% fetal calf serum (FCS) and antibiotics at 37°C in humidified 5% carbon dioxide in air. Subculture was achieved using trypsin-EDTA. Anthracycline (Pharmacia) stock solutions, (1 mg $ml^{-1}$ Hanks' balanced salt solution) were frozen at −20°C.

### *Confocal microscopy*

Sensitive and resistant MGH-U1 cells were seeded into 60 mm Petri dishes and reincubated overnight, allowing the cells to adhere. Two hours before microscopy, the medium was changed to HEPES-buffered DMEM with 10 $\mu g\ ml^{-1}$ of anthracycline with or without verapamil 25 $\mu g\ ml^{-1}$. Cell viability after confocal microscopy was confirmed by trypan blue exclusion (0.02% w/v).

Confocal microscopy was performed using the Leica TCS 4D system, the fibre optic laser emitting at 488 nm. Cells were imaged in the incubation medium using a ×50 water immersion lens. Consistent images were obtained on three separate occasions using identical incubation conditions. Pinhole and electronic variables were kept constant throughout.

### *MTT cytotoxicity studies*

Cytotoxicity experiments were performed using the MTT assay (Freshney, 1994). Cells were seeded in 96-well microtitre plates and exposed to drug at 37°C for 1 h on the following day. Five days later, the plates were incubated with MTT (0.2 mg $ml^{-1}$, 250 $\mu l$ per well) for 4 h, treated with dimethyl sulphoxide (DMSO) and viable biomass determined on a Dynatech MR 5000 plate reader.

### *Spectrofluorimetry studies*

Spectrofluorimetry experiments were carried out at drug concentrations of 10 $\mu g\ ml^{-1}$ using a Perkins-Elmer LS-5B scanning spectrofluorimeter.

### Results

Confocal microscopy uses dual-pinhole optics and raster pattern scanning to produce high definition, fluorescence-based images, enabling intact, viable cells to be visualised as a series of slices. Figure 1a shows the typical, predominantly nuclear epirubicin distribution in sensitive MGHU-1 cells. In contrast, Figure 1b shows punctate cytoplasmic and perinuclear epirubicin distribution in the MDR MGHU-1 cells. These results correspond with published results using other conventional anthracyclines such as daunorubicin or doxorubicin (Coley *et al.*, 1993; Gervasoni *et al.*, 1991).

Correspondence: PM Duffy, Department of Urology, Southampton General Hospital, Southampton SO16 6YD, UK
Received 25 August 1995; revised 19 April 1996; accepted 25 April 1996

Figure 2a shows idarubicin distribution in sensitive MGHU-1 cells. Unlike other anthracyclines in sensitive cells, idarubicin fluorescence appears predominantly perinuclear and cytoplasmic. In some cells an area of intense cytoplasmic drug fluorescence is visible, possibly representing the Golgi apparatus. There is relatively little nuclear drug fluorescence.

Figure 2b demonstrates idarubicin distribution in MGHU-1-resistant cells. Although this specimen shows reduced idarubicin fluorescence, drug distribution remains similar to the sensitive cells.

Addition of 25 $\mu$g ml$^{-1}$ of verapamil to the idarubicin solution increases drug fluorescence in the resistant cells, but appears to make no difference to the distribution of idarubicin fluorescence in either sensitive (Figure 3a) or resistant (Figure 3b) MGHU-1 cells. MTT cytotoxicity studies confirm the P-gp-expressing subline to 100-fold more resistant to idarubicin (Figure 4). Addition of 25 $\mu$g ml$^{-1}$ of verapamil reduces this resistance by a factor of ten.

DNA-mediated fluorescence quenching demonstrates that idarubicin fluoresces more strongly in free solution than the related anthracyclines epirubicin and doxorubicin (Figure 5). Between DNA concentrations of 0.02–0.06 mg ml$^{-1}$, idarubicin demonstrates a greater degree of fluorescence quenching. At greater concentrations, however, the pattern of quenching appears the same.

### Discussion

These results contrast with studies performed on conventional anthracyclines by ourselves and others. Using doxodauno- and epirubicin, nuclear drug fluorescence has been associated with sensitivity, and cytoplasmic fluorescence with resistance (Coley *et al*., 1993; Gervasoni *et al*., 1991). Additionally, the morpholinyl-substituted analogue of doxorubicin (MR-DOX) is known to retain activity in MDR cells, and high levels of nuclear MR-DOX fluorescence in MDR cell lines have been demonstrated (Coley *et al*., 1993).

Although the precise antineoplastic mechanism of action of anthracyclines is still debated, current evidence suggests that these agents intercalate DNA. They may stabilise the topoisomerase–DNA cleavable complex or inhibit DNA helicase activity, thereby reducing replication and transcrip-

**Figure 1** Confocal micrograph of (a) sensitive and (b) resistant MGH-U1 cells incubated for 2 h in 10 $\mu$g ml$^{-1}$ epirubicin, $\times$50 water immersion objective. Grey-scale image proportional to fluorescence intensity.

**Figure 2** Confocal micrograph of (a) sensitive and (b) resistant MGH-U1 cells incubated for 2 h in 10 $\mu$g ml$^{-1}$ idarubicin, $\times$50 water immersion objective. Grey-scale image proportional to fluorescence intensity.

**Figure 3** Confocal micrograph of (a) sensitive and (b) resistant MGH-U1 cells incubated for 2 h in 10 $\mu$g ml$^{-1}$ idarubicin and 25 $\mu$g ml$^{-1}$ verapamil, ×50 water immersion objective. Grey-scale image proportional to fluorescence intensity.

**Figure 4** MTT cytotoxicity assay of sensitivity to idarubicin of parental and MDR MGH-U1 cells, with and without verapamil 25 $\mu$g ml$^{-1}$. Mean viable biomass ±s.e.m. ×2.576. (-■-), sensitive; (-□-), sensitive and verapamil; (-◆-), resistant; (-△-), resistant and verapamil.

**Figure 5** Anthracycline–calf thymus DNA fluorescence quenching. Excitation wavelength = 488 nm. (- - -), epirubicin (10 $\mu$g ml$^{-1}$); (– – –), doxorubicin (10 $\mu$g ml$^{-1}$); (——), idarubicin (10 $\mu$g ml$^{-1}$).

tion (Plosker and Faulds, 1993). As idarubicin is widely regarded as more effective against MDR cells than older anthracyclines (Berman and McBride, 1992), it is curious that nuclear idarubicin fluorescence should appear diminished in both sensitive and MDR cells.

These anomalous findings do not correlate with significantly altered fluorescence quenching. Lankelma *et al.* (1991) demonstrated 95% fluorescence quenching with daunorubicin and calf thymus DNA. Our own work with doxorubicin, epirubicin and idarubicin shows only minor differences in fluorescence quenching between idarubicin and the two conventional anthracyclines and does not explain the striking differences in nuclear fluorescence observed with the confocal microscope.

These results are also relevant to our understanding of the phenomenon of MDR. Verapamil dramatically increases the nuclear uptake and cytotoxicity of conventional anthracyclines in MDR cells (Coley *et al.*, 1993; Michieli *et al.*, 1994). Our own MTT cytotoxicity studies confirm that addition of verapamil to idarubicin preparations increases idarubicin cytotoxicity in the MDR MGH-U1 cell line (Figure 5). However, confocal microscopy demonstrates that the addition of verapamil to idarubicin does not restore nuclear drug fluorescence in either the parental or MDR cell line, suggesting that substantial nuclear drug presence may not be a prerequisite for effective anthracycline cytotoxicity in sensitive cells, or for overcoming MDR.

Considerable work remains to be done, both on the putative mechanism of action of anthracyclines, and on the fundamentals of MDR and MDR reversal. We believe that our results are relevant to the continuing study of these mechanisms and that it is premature to assume that nuclear drug fluorescence necessarily correlates with cytotoxicity.

**Acknowledgements**
We gratefully acknowledge the award of a Research and Development grant (No. 061956291405) by Wessex (Southwestern) Regional Health Authority, and additional financial assistance to enable us to pursue our confocal studies from Pharmacia (UK).

### References

BERMAN E AND MCBRIDE M. (1992). Comparative cellular pharmacology of daunorubicin and idarubicin in human multidrug-resistant leukaemia cells. *Blood*, **12**, 3267-3273.

COLEY HM, AMOS PR, TWENTYMAN PR AND WORKMAN P. (1993). Examination by confocal fluorescence imaging microscopy of the subcellular localisation of anthracyclines in parent and multidrug resistant cell lines. *Br. J. Cancer*, **67**, 1316-1323.

DUFFY PM, HAYES MC, GATRELL SKE, COOPER A AND SMART CJ. (1996). Determination and reversal of resistance to epirubicin intravesical chemotherapy. A confocal imaging study. *Br. J. Urol.*, **77**(6), 824-829.

FLOYD JW, LIN C AND PROUT GR. (1990). Multi-drug resistance of a doxorubicin-resistant bladder cancer cell line. *J. Urol.*, **144**, 169-171.

FRESHNEY RI. (1994). *Culture of Animal Cells. A Manual of Basic Technique*. pp. 296-298. Wiley-Liss: New York.

GERVASONI JE, FIELDS SZ, KRISHNA S, BAKER MA, ROSADO M, THURAISAMY K, HINDENBURG AA AND TAUB RN. (1991). Subcellular distribution of daunorubicin in P-glycoprotein-positive and -negative drug-resistant cell lines using laser-assisted confocal microscopy. *Cancer Res.*, **51**, 4955-4963.

LANKELMA J, MÜLDER HS, VAN MOURIK F, WONG FONG SANG HW, KRAAYENHOF R AND VAN GRONDELLE R. (1991). Cellular daunomycin fluorescence in multidrug resistant $2780^{AD}$ cells and its relation to cellular drug localisation. *Biochim. Biophys. Acta*, **1093**, 147-152.

MICHIELI M, DAMIANI D, MICHELUTTI A, CANDONI A, MASOLINI P, SCAGGIANTE B, QUADRIFOGLIO F AND BACCARANI M. (1994). Restoring uptake and retention of daunorubicin and idarubicin in P170-related multidrug resistance cells by low concentration D-verapamil, cyclosporin-A and SDZ PSC 833. *Haematologica*, **79**, 500-507.

MOSCOW JA, SCHNEIDER E AND COWAN KH. (1993). Multidrug resistance. In *Cancer Chemotherapy and Biological Response Modifiers*, Pinedo HM, Longo DL, Chabner BA (eds), Annual 14, pp. 98-117. Elsevier Science: Oxford.

PLOSKER GL AND FAULDS D. (1993). Epirubicin. A review of its pharmacodynamic and pharmacokinetic properties and therapeutic use in cancer chemotherapy. *Drugs*, **45**, 788-856.

ROEPE PD. (1992). Analysis of the steady-state and initial rate of doxorubicin efflux from a series of multi-drug resistant cells expressing different levels of P-glycoprotein. *Biochemistry*, **31**, 12555-12564.

SCHEFFER GL, WIJNGAARD PLJ, FLENS MJ, IZQUIERDO MA, SLOVAK ML, PINEDO HM, MEIJER CJ, CLEVERS HC AND SCHEPER RJ. (1995). The drug resistance-related protein LRP is the human major vault protein. *Nature Med.*, **1**, 578-582.

ZAMAN GJR, FLENS MJ, VAN LEUSDEN MR, DE HAAS M, MÜLDER HS, LANKELMA J, PINEDO HM, SCHELPER RJ, BAAS F, BROXTERMAN JH AND BORST P. (1994). The human multidrug resistance-associated protein MRP is a plasma membrane drug-efflux pump. *Proc. Natl Acad. Sci. USA*, **91**, 8822-8826.